# Evolution of Quality Parameters and Bioactivity of *Actinidia chinensis* cv. Sungold (Kiwifruit) Slices Subjected to Different Drying Conditions Storage for 4 Months

**DOI:** 10.3390/foods13132100

**Published:** 2024-07-01

**Authors:** Sicari Vincenzo, Mincione Antonio, Romeo Rosa, Pino Roberta, Conforti Filomena, Loizzo Monica Rosa

**Affiliations:** 1 Department of Agraria, University Mediterranea of Reggio Calabria, University Citadel, Locality Feo di Vito, 89124 Reggio Calabria, RC, Italy; vincenzo.sicari@unirc.it (S.V.); amincione@unirc.it (M.A.); rosa.romeo@unirc.it (R.R.); 2 Department of Pharmacy, Health and Nutritional Sciences, University of Calabria, 87036 Rende, CS, Italy; robertapino95@gmail.com (P.R.); filomena.conforti@unical.it (C.F.)

**Keywords:** hot air drying, drying quality, antioxidant activity, phenolic compounds, texture, organic acids

## Abstract

The present study aimed to investigate the impact on nutritional and functional properties of dried kiwifruit (*Actinidia chinensis* cv. Sungold) slices during conservation for 120 days in sealed containers in the dark at 25 °C. For this purpose, kiwifruits slices were dried at two different temperatures, 40 and 55 °C, for 30 and 25 h, respectively. Fresh and dried kiwi slices were analyzed for their pH, activity water, total solid soluble (TSS), color, titratable acidity, total phenols (TPC) and flavonoids content (TFC), organic acids, and radical scavenging activities. Analysis carried out on the dehydrated samples showed a good aptitude of kiwi material towards the drying process. Particularly, it has been observed that the drying treatment at low temperature helped to preserve the nutraceutical properties of the fruits. In fact, samples treated at 40 °C (KLT) showed at day 0 (T0) the highest TPC and TFC with values of 979.42 Gallic Acid Equivalents (GAE)/100 g of dried weight (dw) and 281.84 mg catechin equivalents (CTE)/100 g dw even if compared with fresh kiwi slices sample (FKF). Moreover, KLT also exhibited the highest values of antioxidant activity (1657 mmol Trolox/100 g dw). After 120 days storage, all dried samples showed a high ascorbic acid content (429–339 mg/100 g dw fruits) and only a slight variation of physicochemical parameters. Textural Parameters (hardness, springiness, cohesiveness, gumminess, and chewiness), apart from resilience results, showed significant differences between kiwifruit dried at 55 °C and at 50 °C (KLT and KHT, respectively). Color and aroma intensity were the main sensory descriptors with higher scores.

## 1. Introduction

Kiwi is a fruit native to China. At the end of the 20th century, it arrived in Europe and found diffusion especially in Italy, which today represents one of the largest exporters together with New Zealand [1].

The most cultivated kiwifruits belong to the *Actinidia deliciosa* cv. Hayward species. However, an increase in the consumption of golden-fleshed kiwifruit (*Actinidia chinensis* spp.) has recently been observed. These fruits have bright yellow pulp surrounded by a glabrous bronze skin. This fruit differs from the Hayward kiwi as it has a sweet flavor and a tropical taste [2,3]. Gold kiwis are consumed fresh without removing the covering peel, either in the form of juice or as ingredients in various recipes, both sweet and savory [3]. Kiwifruits are rich in vitamin C, E, polyphenols and flavonoids, iron, potassium, and fiber [4,5,6,7]. Numerous studies have shown that the content of bioactive compounds in these fruits means that their consumption helps prevent pathologies such as diabetes, cardiovascular diseases, and cancer [8].

Kiwifruits are classified as climacteric fruits, which means that it continues to ripen even post-harvest. This probably determines a rather short shelf life, in addition to its high moisture content (~80%). The quality of the fruit depends on numerous factors: growing area, climatic and cultivation conditions, stage of ripeness at harvest, and storage conditions [9].

Mild technologies are able to maintain the freshness of the food product, including its organoleptic properties, and, at the same time, to prolong its shelf life [10]. Moreover, these technologies, including low temperature operating processes, are advantageous for maintaining adequate levels of micronutrients [11,12].

Dehydration is one of the best fruit preservation methods [13]. In Italy, this practice is widely applied to sliced plums, apricots, and apples. Dehydrated fruit is characterized by interesting health properties and ease of use compared to the fresh product [14,15].

The market size for snacks rich in vitamins, dietary fibers, and minerals has grown significantly in the last 5 years. The global dried fruit snacks market is expected to grow with a compound annual growth rate (CAGR) of about 7.1% during the period 2023–2030 [16]. In this context, the dried kiwifruit market size is estimated to grow with a CAGR of 5.9% during the period 2022–2027 [17].

Several research works investigated the impact of different drying methodologies on kiwifruits slices by applying convective, microwave, and freeze-drying processes [5,18,19,20,21,22,23,24,25,26,27,28,29,30,31,32,33,34]. However, all the works concern the Hayward cv. except Diamante et al. [29], who evaluated the impact of drying process (60, 80, and 100 °C with air velocity of 0.20 m/s at ambient humidity) on Hayward and Sungold kiwifruit slices.

This work aimed to highlight, for the first time, the impact on nutritional and functional properties of dried Sungold cv. kiwifruit slices during conservation for 120 days in sealed containers in the dark at 25 °C. For this purpose, kiwifruits slices were dried at two different temperatures, 40 and 55 °C, for 30 and 25 h, respectively.

Chemical-physical parameters, total phenols, flavonoids and ascorbic acid contents, radical scavenging potential, sensory, and texture analysis were assessed.

## 2. Materials and Methods

### 2.1. Dried Kiwifruit Preparation

**Figure 1:**
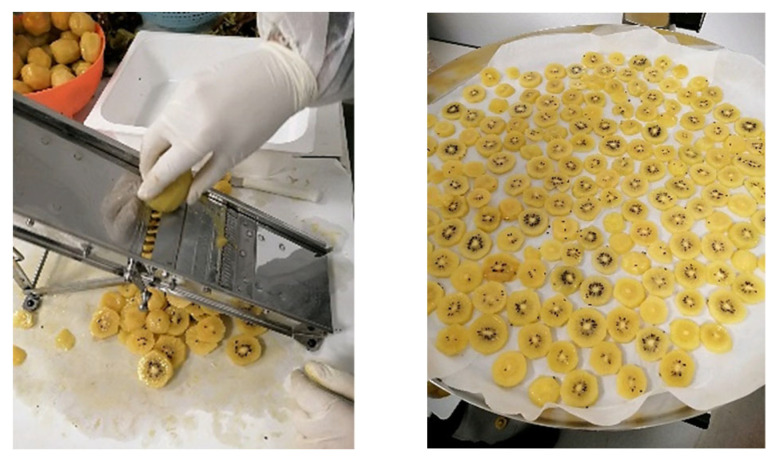
Kiwifruits cut into slices and distributed into trays in a single layer.

Kiwifruits (*Actinidia chinensis* cv. Sungold) were obtained from a farm in Montalto Uffugo (Cosenza, Italy) in February 2023. Homogeneous (per caliber) kiwifruit samples (15 kg) were washed with a sodium hypochlorite solution, then peeled and cut vertically to their axis into 7 mm thick cylindrical slices using a mechanical cutter (Figure 1).

The drying process was carried out in a convective dryer (model “Scirocco”, Società Italiana Essiccatoi, Milan, Italy) set at two different temperatures: 40 °C and 55 °C.

The kiwi slices were distributed in trays in a single layer and immediately dried in a tangential air flow cabin equipped with automatic devices for controlling the temperature and humidity of the air. The air flows tangentially to the fruit (1840 m^3^/h), while a recycling system allows the exhaust gases to be mixed with fresh air. The air speed was 1.19 m/s, while the relative humidity was 77%. These parameters were monitored by using a microprocessor controller. The fresh air was recycled by a fan powered by a motor.

The process was carried out until a final humidity of ~20% compared to the initial one was reached, i.e., for 30 and 25 h for 40 and 55 °C, respectively.

**Figure 2:**
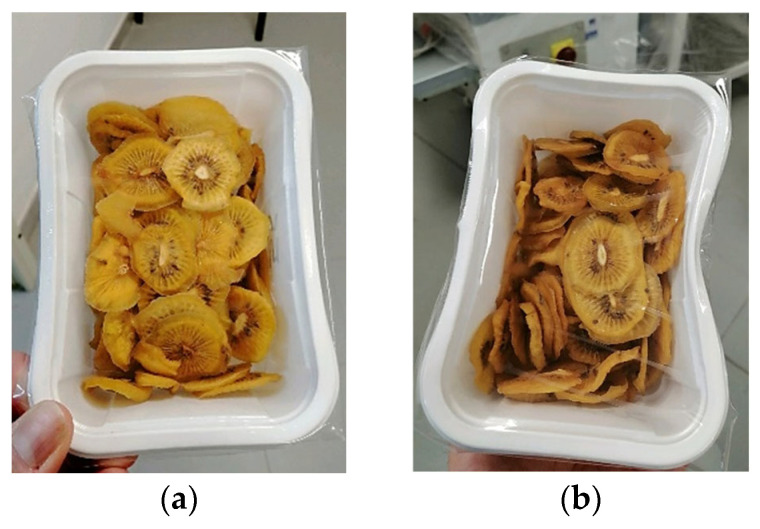
Kiwi dried samples stored in sealed containers at (**a**) 40 °C (KLT) and (**b**) 55 °C (KHT).

Kiwi slices dried at 40 °C for 30 h were identified as KLT whereas slices dried at 55 °C for 25 h were identified as KHT. Dried kiwifruit slices (KHT and KLT) were stored in sealed containers (Figure 2) in the dark at 25 °C and analyzed after 0, 30-, 60-, 90, and 120-days storage.

### 2.2. Preparation of Extracts

For determination of chemical parameters, kiwifruits were homogenised in a blender (Electrolux E4CB1-6ST, Stockholm, Sweden) then 5 g of the sample were added to 50 mL of distilled water and centrifuged using Nüve NF 1200R (Saracalar Kümeevleri, Ankara, Turkey) (5000 rpm, swing-out rotor 160 mm, for 10 min). Supernatant solution was filtered through Whatman no. 1 filter paper and analyzed.

For the estimation of total phenols (TPC) and flavonoids (TFC), homogenized kiwifruits (5 g) were added to 25 mL of a methanol: water (80:20, *v*:*v*) solution and then centrifuged at 5000 rpm for 10 min according to the method of Sicari et al. [35]. Supernatant collected was filtered through syringe filters (Ch 0.45 μm Chromafil RC-45/25) and used for the for subsequent analyses.

### 2.3. Physico-Chemical and Colorimetric Analysis

The moisture content was determined following AOAC procedure [36]. Kiwifruits were accurately weighed before and after oven drying (Binder WTC, Tutlingen, Germany) until reaching the constant weight.

Titratable acidity (TA) was measured according to the AOAC method and expressed as g of citric acid/100 g dw whereas pH measurement was performed by using a pH meter Crison GLP-21 (Crison Instrument, Barcelona, Spain). Water activity (a_w_) was measured using an Aqualab LITE hygrometer (Decagon Devices Inc., Washington, DC, USA) [37,38].

A digital refractometer PR-201α (Atago, Milan, Italy) was used for total solid soluble content (TSS). Results were expressed as °Brix.

Ten kiwifruits were used for the CIELab color parameters determination (L, a*, and b*) using a colorimeter (CR-400, Konica Minolta, Osaka, Japan). Fruits were measured on three different points on the cut surface for a total of thirty measures for each treatment. The Chroma (C), Hue angles range (h), ΔE, and browning index (BI) parameters were calculated [35].

### 2.4. Evolution of Organic Acids during Storage

Analysis of kiwifruit extracts was performed using a Knauer (Asi Advanced Scientific Instruments, Berlin, Germany) system equipped with two pumps (Smartiline Pump 1000), a Rheodyne injection valve (20 µL), and a photodiode array detector UV/VIS. Organic acids was separated on a Knauer RP C18 column (250 mm × 4.6 mm, 5 µm) in isocratic mode with a mobile phase of 0.2 M KH_2_PO_4_, a flow rate of 0.6 mL/min, and an injection volume of 20 µL. Detection was carried out at 210 and 245 nm. External standard calibration curves were used for the identification and quantification of organic acids. Five injections were made for each calibration level. For the linear regression of the curves of external calibration standards, R^2^ values were between 0.997 and 0.999. Data processing was carried out with the support of Clarity Software 6.2 (Chromatography Station for MS Windows) and results were expressed as mg 100/g dw.

### 2.5. Total Phenols and Flavonoids Content and Evaluation of Bioactive Compounds Evolution during Storage

For the evaluation of TPC, kiwifruit extract was mixed with Folin–Ciocalteu reagent and Na_2_CO_3_. Results were expressed as mg Gallic Acid Equivalents (GAE)/100 g of dried weight (dw) [5]. The determination of TFC was conducted by using the procedure previously described by Zhang et al. [9]. Results were expressed as mg catechin equivalents (CTE)/100 g dw. The impact of drying processes applied to kiwifruits was investigated by first order mathematic kinetic model as previously described [35].

### 2.6. Radical Scavenging Activity

The radical scavenging activity was assessed using two different in vitro assays: 1,1-diphenyl-2-picryl hydrazine (DPPH) and 2,2’-azinobis-3-ethylbenzothiazoline-6-sulfonate (ABTS). The DPPH test was conducted following the procedure previously reported by Brand-Williams et al. [39]. Briefly, DPPH methanol solution was added to the kiwifruit extract. The absorbance was measured after 15 min at λ = 515 nm. Trolox was used as a standard antioxidant and samples activity was expressed as mmol of Trolox/100 g dw.

The ABTS radical test was carried out as previously described [40]. Briefly, a solution of ABTS radical was diluted (1:80) with ethanol to give an absorbance of 0.70 at λ = 734 nm. An aliquot of extract was added to ABTS solution, and the absorbance was measured at 734 nm. Trolox was used as a standard antioxidant and samples activity was expressed as mmol of Trolox/100 g dw.

### 2.7. Firmness and Texture Analysis

Texture Profile Analysis (TPA) rheological analyses were performed with a TA-XT Plus Texture Analyzer (Stable Micro Systems Ltd., Godaming, UK) and computed with companion software (Exponent 6.1.4.0, Stable Micro Systems Ltd., Godaming, UK (Figure S2). Texture Profile Analysis test was performed using a 100 mm compression platen (P/100 compression platen probe, Stable Micro Systems Ltd., Godaming, UK probe on single samples with the following operational parameters: pre-test speed: 1.50 mm/s; test speed: 1.50 mm/s; post-test speed: 5.00 mm/s; distance: 3.0 mm; trigger force: 5.0 g; data acquisition rate: 200 pps. For each sample, ten repetitions were carried out. TPA textural parameters (hardness, springiness, cohesiveness, gumminess, chewiness and resilience) data were expressed as mean values; means were further analyzed by one-way ANOVA and Tukey’s test, at 5% probability, using statistical software (IBM SPSS Statistics for Windows, Version 20, IBM Corp., Armonk, NY, USA).

### 2.8. Sensorial Analysis

Quantitative descriptive sensory analysis (QDA) of samples was carried out by a trained panel composed by 10 people (5 males, 5 females, 22–40 years old, regular consumers of the product, recruited among students and staff of the “Mediterranea” University of Reggio Calabria, Italy). Samples were served randomly in sensory booths in the Food Sensory laboratory of the “Mediterranea” University of Reggio Calabria. Judges rated samples on a 6-point structured scale for appearance, olfactory, taste, and textural descriptors (Table 1). Minimum score (0) indicated the absence of the attribute, while 5 indicated a very intense attribute.

### 2.9. Statistical Analysis

All experiments were performed in triplicate. The effects of the treatment methods and storage time were evaluated by statistical analysis of variance (one-way ANOVA) using IBM SPSS Statistics software (version 21.0, IBM, Armonk, NY, USA). All data were presented as mean values and standard deviations (*n* = 3). Tukey’s multiple range test was used to evaluate differences among values, and the statistical significance was defined as *p* < 0.05.

## 3. Results and Discussion

### 3.1. Quality Parameters

Two different drying temperatures were applied in this study and compared with fresh kiwi fruits to evaluate the impact of processing on dried kiwifruit slices. One (40 °C) should allow greater retention of bioactive compounds, while the other (55 °C) was selected to increase the non-enzymatic browning reaction and study its effects on the levels of polyphenols, flavonoids, and antioxidant properties.

FKF showed a penetrometer resistance of 120.92 N and a sugar content of 14.50 °Brix.

The moisture content of the fresh kiwifruit (FKF) was 81.35% g/100 g (wb), which was close to the values reported by Kaya et al. [14] and by Simal et al. [26], which were 81% and 82% wb, respectively.

The dried samples all showed moisture contents under 20%, established as the end point of drying, according to conservation criteria, since this value allowed good preservation while still maintaining good final physical and chemical properties [41].

Furthermore, as the temperature increased, the moisture content diminished, and the samples became more dehydrated.

The TSS are represented by sugars, acids, vitamins, some minerals, and other soluble solids, and are essential indicators of sensory quality. Kiwi slices samples were analyzed fresh (FKF) and after the drying process at 40 (KLT) and 55 °C (KHT), respectively.

Table 2 shows the values relating to a_w_, pH, TSS, TA, and BI. The a_w_ of dried kiwifruits slices was significantly reduced compared to the fresh sample, with values of 0.99, 0.45, and 0.50 for FKF, KLT, and KHT, respectively. The a_w_ of both KLT and KHT samples was significantly reduced in comparison with the FKF, thus allowing the preservation of the food, since low values of a_w_ no microbial, chemical, and enzymatic reactions occur [26]. Our results resulted in agreement with those found in literature for fresh kiwi [9,25,41]. In particular, Correia et al. [25] applied temperatures from 50 to 80 °C to kiwifruits (cv. Hayward) and found a_w_ in the range 0.75–0.66 for 50 and 80 °C, respectively, and a moisture content from 19.89 and 10.01 for the same applied treatment. It is interesting to note that in our case. even at lower temperature (40 °C), the a_w_ is lower (0.45).

The pH values increased slightly after the drying treatment (from 3.19 to 3.56 and 3.52 for FKF, KLT, and KHT, respectively). A similar trend was also found for TA, where values of 1.51, 0.74, and 0.76 g/100 g for FKF, KLT, and KHT, respectively, were recorded.

Food color represents one of the parameters for choosing one food rather than another one even before taste, hence the need to study the impact of processing on colorimetric parameters. Table 3 shows the CIELab color coordinates for dried kiwifruit slices (KHT and KLT). The coordinates for FKF were approximately 58.57, 1.19, and 15.34 for brightness (L), green (a*), and yellow (b*), respectively. Generally, higher C and *h* values were evidenced in dried samples with values of 24.18, and 1.48 vs. 20.17, and 1.41 for KHT and KLT, respectively (Table S1). The L parameter appeared to increase slightly with drying at 55 °C, while at 40 °C it remained similar to the value found for FKF sample. Conversely, a* parameter tended to increase, thus indicating that the intensity of the green color was reduced with the drying process regardless of the applied temperature. This finding could be related to non-enzymatic browning phenomena, which make the kiwifruit slices greenish as the drying temperature increases. As regards the b*, it was observed that the drying process caused an increase of this parameter compared to the KFK with values of 23.45 and 15.34 for KHT and FKF, respectively. This may also be linked to the effect of high temperature on proteins and carbohydrates (Maillard reaction). With regard to the h parameter, a slight decrease was observed during storage independently by the temperature applied in drying process. Previously, Maskan [28] reported that drying process changed L, a*, and b*, causing a color shift towards the darker region. Among hot air, microwave (MW), and hot air-MW finish drying, the h parameter was more influenced by MW drying process.

No significant differences were recorded between L and C parameters measured at T0 and T120. However, ΔE value after 120 days of storage evidenced that samples treated at higher temperature (KHT) had double ΔE compared to the KLT (3.16 vs. 1.08, respectively). Our data on L parameters disagree with those reported by Diamante et al. [29], who recorded a decrease of this parameter in gold kiwifruits. The L parameter in our fresh sample (FKF) was 1.29-times greater than the data reported for the Hayward kiwi by Correia et al. [25] (L = 45.34). Moreover, the impact of different temperatures on this parameter resulted in an L variation from 47.45 to 52.43 at 50 and 80 °C, respectively. An opposite trend was observed by Izli et al. [5], who found a reduction in the L parameter as function of the temperature increase from 60 to 80 °C.

Previously, Zhang et al. [42] evaluated the shelf-life of fresh *A. deliciosa* kiwifruits for 80 days using different temperatures. Authors found an increase in ΔE parameter during storage with values from 1.15 to 9.42 at 3 and 11 days, respectively, with kiwifruits under dynamic temperature conditions (5 °C for 5 days → 20 °C).

No significant differences in the parameter a* were found between the two samples dried at different temperatures and therefore in the red color of the matrix. An opposite trend was observed by Simal et al. [27]. On the contrary, b* was highest in kiwifruits dried at the highest temperature with values of 18.74 and 23.11 for KLT and KHT at T0, respectively. In this case, our data disagree with those previously reported by other authors. The different findings on color parameters between the studies could depend on the fact that our operating temperature conditions are much milder than other experimental approaches (40 and 55 °C vs. 60, 80, and 100 °C, respectively).

**Figure 3:**
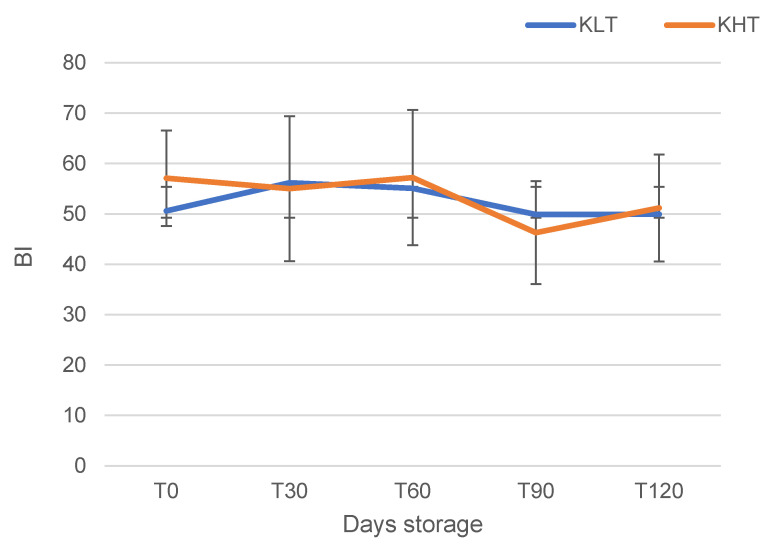
Browning index (BI) evolution during storage of kiwifruit slices dried at 40 °C (KLT) and 55 °C (KHT).

At T0, KHT showed the highest browning index (BI) value (57.09) whereas no significant differences were recorded after 120 days storage (49.92 and 51.19 for KLT and KHT, respectively) (Figure 3). Considering that the browning process of fruits and vegetables is related to the concentration of phenolic compounds, the activity of polyphenol oxidase (PPO), as well as the temperature, pH, and availability of oxygen in the tissues, it is conceivable that the different trend of the BI, which occurred in dried samples, can be explained through the different impact that temperature had on enzymatic reactions. In fact, at 40 °C, the enzymatic reactions are slower than those that occur when the temperature is increased to 55 °C [41].

As expected, the total color difference increases significantly with increasing temperature from 40 to 55 °C, since higher temperatures favor browning reactions due to polyphenol oxidase and the presence of oxygen [41].

The TSS values in the KLT samples show an increase from 2.43 to 2.47 °Brix at T0 and after T1210, while the values of TA decrease from 0.74 to 0.58 g citric acid/100 g dw. Our data are in agreement with those found by Zhang et al. [42], where TSS increased from approximately 9% to 15% while TA decreased from approximately 1.5% to 1.1% during storage.

### 3.2. Organic Acid Evolution during Storage

The organic acid values were determined by Ultra-High-Performance Liquid Chromatography (UHPLC). These compounds are important constituents in determining sensory quality characteristics. In kiwi, during storage and ripening, the titratable acidity undergoes slight modifications, often related to the place of cultivation [43,44]. Until now, very little information is available on the relationships between organic acids and other kiwi constituents and on the factors that influence them in the fruit [45]. Ascorbic, citric, malic, tartaric, and oxalic acids were identified (Table 4). Among them, citric acid represents the most abundant organic acid in FKF, with a value of 2215.47 mg/100 g dw, followed by ascorbic acid (957.22 mg/100 g dw). A similar concentration was found for both malic and tartaric acids (638.53, and 674.98 mg/100 g dw, respectively).

The analyses carried out on dried samples of kiwifruit slices evidenced that citric acid concentration was higher KLT in comparison to KHT with value at T0 of 4664.67 and 2008.12 mg/100 g dw, respectively. An opposite trend was observed for oxalic acid that are higher in KHT when compared to KLT (at T0 of 99.67 and 27.49 mg/100 g dw, respectively).

The quality of the dried kiwi slices was also evaluated by monitoring the ascorbic acid content according to the storage period. Ascorbic acid exerts a series of positive effects on human health, including reduction of incidence of cancer, high blood pressure, tissue regeneration, etc. [46].

As expected, the ascorbic acid content decreases with increasing drying temperature, with values of 954.22, 1009.74, and 444.95 mg/100 g dw for FKF, KLT and KHT, respectively. This evidence agrees with those found previously by Vega-Galvez et al. [47] and Santos and Silva [48], who demonstrated how the loss of ascorbic acid due to the application of high temperatures can be associated with the ascorbic acid thermo-sensitivity character and the easily degradable structure that undergoes oxidation to dehydroascorbic acid [30,31]. However, a loss of ascorbic acid can also be observed at lower temperatures such as 40 °C, but for more prolonged exposures.

Our data are in agreement with Kaya et al. [30], who found a retention of ascorbic acid of 117.65 and 27.47 mg/100 g for kiwi slices dehydrated at 35 and 65 °C, respectively. In contrast, Diamante et al. [29] did not evidence significant changes in the ascorbic acid content of fresh and dried green and gold kiwifruits when the drying procedure was conducted at 60 and 80 °C. However, if the temperature rises to 100 °C, an approximately 19% loss of ascorbic acid content was observed in both green and gold kiwifruits. These differences may be accounted for by the different drying time (12 h) for the study of Kaya et al. [30] work and about 6 h in this last case. Previously, Tepe et al. [31] reported a loss of ascorbic acid in kiwifruits of −57.27, −59.90, and −64.22% at 60, 70, and 80 °C, respectively. A higher loss was found by Correia et al. [25], who reported values in the range of −76, and −82% at the end of the drying process carried out at 60 and 80 °C, respectively. A significant reduction in ascorbic acid content also occurred as an effect of storage and this was more pronounced for the KLT sample than for KHT with an estimated percentage loss of −66.33 and −13.63%, respectively, after 120 days of storage.

Movagharnejad and Pouya [18] compared the impact of different drying process (convective tray dryer, microwave dryer, and freeze dryer) on ascorbic acid content and found the major retention of this bioactive occurred when the freeze-drying process was applied (80% of fresh kiwifruits). The effect of freeze drying (FD), hot air drying (HAD), vacuum drying (VD), and hot air–microwave assisted vacuum combination drying (HA–MVD) on Hayward kiwifruits was assessed [22]. The main loss of ascorbic acid was found when HAD was applied (−77.52%), with a value of 249.17 mg/100 g.

A lower content in ascorbic acid was found by Zhang et al. [42] in fresh Hayward kiwifruits with the value of 93.3 mg/100 g. This bioactive undergoes a significant reduction during storage, reaching 64.3 mg/100 g after 11 days of storage at 20 °C.

### 3.3. Total Phenol and Flavonoid Content and Radical Scavenging Activity

The TPC in fresh kiwifruit was found to be equal to 941.79 mg GAE/100 g dw (Table 5). This value was higher than that reported by Leontowicz et al. [49] and Gümüşay et al. [32], who found TPC in the range 262.66–540.00 mg GAE/100 g dw; similar values were found by Chin et al. [24].

The effect of the drying process on TPC and TFC was studied, and the results are summarized in Table 5. Drying temperature influenced the TPC in kiwifruit slices; at T0, values of 979.42 and 562.04 mg GAE/100 g dw were recorded for KLT and KHT, respectively. It is interesting to note that the TPC of KLT is quite similar to the FKF sample at T0 (941.79 mg GAE/100 g dw). This could be due to the lower water loss during the drying treatment at a lower temperature [50,51,52]. The decrease in TPC as consequence of heat treatment has been reported by other authors [14,53]. During the storage of dried kiwi slices, a clear decrease after the first 30 days of storage was observed in both samples, with values of 650.54 and 472.27 mg GAE/100 g dw for KLT and KHT, respectively. In general, the KLT sample suffered a greater loss of TPC (−59.35%) compared to the value recorded at T0 compared to KHT (−22.39%). For TFC, values of 260.19, 281.84, and 169.07 mg CTE/100 dw were recorded for FKF, KLT, and KHT, respectively. Also, for TFC, while the concentration of these compounds in the slices dried at 40 °C remained unchanged, a slight decrease was observed for the samples dried at 55 °C. During storage, these values were further reduced, reaching a concentration of 113.93 and 102 mg CTE/100 g dw for KLT and KHT, respectively. This reduction is more accentuated in samples dried at 55 °C compared to those obtained by applying the drying temperature of 40 °C. In fact, a loss of −59.57 and −39.20% for KHT and KLT, respectively, was recorded at T120.

A loss of TPC in kiwifruits of −59.43%, −54.72%, and −53.61% at 60, 70, and 80 °C, respectively, was found by Tepe et al. [39]. A significant loss in TPC was found by Correia et al. [25], who reported a decrease from −80 to −93% in kiwifruits dried at temperatures of 60 to 80 °C, respectively.

Previously, Izli et al. [5] reported the effect of convective (60, 70, and 80 °C), microwave (120 and 350 W), and freeze-drying methods on the TPC of kiwi slices and found a decrease ranging from −5 to −49% following drying treatments. Kiwi slices dehydrated by freeze-drying process exhibited the highest TPC, with value of 361.38 mg GAE/100 g dw; on the contrary, the application of microwave at 120 W determined the lowest TPC value (193.05 mg GAE/100 g dw). This could be caused by the fact that the lower drying temperatures used with the microwave did not inactivate the oxidative enzymes completely, which then resulted in oxidation of the phenolic substances and, statistically, a lower TPC value. However, temperature is one of the factors affecting the degradation of the phenolic compounds; the other one is drying time, since a longer time causes higher degradation.

Table 5 reported the radical scavenging potential of fresh and dried kiwifruit slices activity evaluated by ABTS and DPPH tests. Regarding DPPH, at T0 it is possible to observe that KLT was more active than FKF with values of 1657.62 and 1195.87 mmol Trolox/100 g dw, respectively. A similar situation was observed by Ozcan et al. [23], who proposed that heating processes destroy the integrity of the cell structure of kiwifruits, thereby promoting the release of phenols during extraction procedure and consequently increasing the antioxidant activity of the extract. Conversely, a lower DPPH radical scavenging activity was found for KHT with values of 926.15 mmol Trolox/100 g dw.

This behavior could be explained by the reduction of TPC after the drying process, which also resulted in a reduction in antioxidant capacity. This situation was also observed by Degirmencioglu et al. [53] for blueberry fruits. However, the radical scavenging potential against DPPH underwent only a slight reduction, with a value at T120 equal to 996.79 and 854.35 mmol Trolox/100 g dw for KLT and KHT, respectively.

In the ABTS assay, the radical scavenging activity at T0 was slightly higher than that recorded for fresh kiwifruits independently by the drying temperature applied with values of 56.05, 64.68, and 67.59 mmol Trolox/100 g dw for FKF, KLT, and KHT, respectively. A positive Pearson’s correlation coefficient was found between TPC and DPPH and ABTS data with R^2^ values of 0.93 and 0.95, respectively, whereas values of 0.88 and 0.80 were found for TFC and the same antioxidant data.

As for other types of fruits, the intrinsic antioxidant activity is related to cultivar and harvesting time [54].

Pal et al. [55] evaluated the effect of fruit harvesting stage on the antioxidant properties in five kiwi cultivars, namely Abbot, Bruno, Allison, Hayward, and Monty.

Generally, kiwifruit exhibited the highest radical scavenging activity at the start of fruit development. In particular, the following trend was observed by comparing the different cultivars, ‘Allison’ > ‘Abbot’ > ‘Bruno’ > ‘Hayward’, in both DPPH and ABTS tests. The relationship between cultivars and radical scavenging potential was also found by Ozen et al. [15], who recorded IC_50_ values of 53.11 and 123.31 for Greenlight and Topstar kiwifruits cultivars, respectively.

The effect of different drying treatments, convection drying (CD), microwave drying (MD) and hybrid drying (HD), on antioxidant activity of kiwifruits slices was investigated and compared with fresh kiwi samples [33]. At the same concentration test, a greater inhibitory activity against DPPH radical was found with the fresh sample, followed by the HD sample (60 °C + 300 W). The reduction of antioxidant activity is related to the destruction of bioactive compounds such as phenols, which further leads to chemical, enzymatic, or thermal decomposition. Moreover, the evidence that the antioxidant potential was higher in HD than in CD and MD could be because partially oxidized polyphenols have better antioxidant activity than non-oxidized polyphenols [56].

Our data are in agreement with the trend observed by Izli et al. [5], who demonstrated how a reduction in antioxidant activity should be observed in dried kiwifruits samples in comparison to the fresh one. Moreover, no significant differences were recorded between convective (60, 70, and 80 °C), and microwave (120 and 350 W) dried samples, with values of 4.71, 5.08, 5.23, 4.42, and 4.66 μmol Trolox/g dw, respectively. In addition, non-thermal freeze-dried kiwi slices (7.94 μmol Trolox/g dw) had notably higher antioxidant capacity when compared to the other dried samples.

### 3.4. Degradation Kinetic of TPC and TFC Content during Storage

The effects of the drying temperatures (40 and 55 °C) used for drying the kiwi slices were determined through the degradation kinetics of the quantified TPC and TFC.

Obtained data evidenced that TPC followed a first order reaction, with R^2^ ranging from 0.9074 to 0.9527, whereas a zero-order reaction was found for TFC with an R^2^ ranging from 0.8543 to 0.9173 (Table 6, Figure S1).

The stability of TPC and TFC during storage was evaluated considering changes in their concentration. Table 6 shows the kinetic parameters (kinetic rate constant (k) and half-life values (t_1/2_)) determined for the thermal degradation of TPC and TFC. The kinetic parameter k is an indicator that predicts the thermal degradation of the phytochemical content, where the lower k value indicates better stability of the analyzed bioactive compounds. However, the values of t_1/2_ allow us to predict the progress of degradation during storage.

As expected, there was a decrease in the percentage of TPC, TFC, and ascorbic acid during storage. In fact, as shown in Table 6, the TPC half-life time values (t_1/2_) were 385.080 and 99.021 for KLT and KHT, respectively. This trend can be explained by the fact that at T0, the TPC was greater in KLT compared to KHT; this reduction is due to the higher drying temperature. Subsequently, in the first 30 days of storage, the concentration of TPC tends to decrease drastically, although remaining at rather high values, in samples treated at a temperature of 40 °C. The same trend was observed for ascorbic acid, while, in the case of TFC, the decrease was not as drastic as in the case of other bioactive phytochemicals.

These results suggest that “mild” drying initially preserves the thermolabile components and avoids major changes in the organoleptic properties of the dried kiwi, for this reason the organoleptic and functional quality of the kiwi slices dried at a temperature of 40 °C is better during the shelf life.

Subsequently, during storage, the phytochemical reactions observed towards TPC, TFC, and ascorbic acid are probably due to the activity of polyphenol oxidases (POO), the enzyme responsible for their oxidation, and this greater activity could be responsible for the decrease in their contents during storage.

Furthermore, as reported in the literature, the decrease or increase of these compounds depends greatly on the temperature during treatment and the drying time [47,56,57].

### 3.5. Texture and Sensory Analysis

Texture profile analysis (TPA) results obtained from kiwi samples are summarized in Table 7.

All TPA results (in hardness, springiness, cohesiveness, gumminess, and chewiness), apart from resilience results, showed a significant difference between KLT and KHT samples, with values sometimes 2-fold higher or more in KLT over KHT samples.

As for the evolution during storage of hardness, gumminess, and chewiness, results showed a steep increase at the 30-days of storage sampling, while cohesiveness showed a steadily decreasing trend. All parameters, however, showed a rather high degree of variation among replicates.

### 3.6. Sensory Analysis

**Figure 4:**
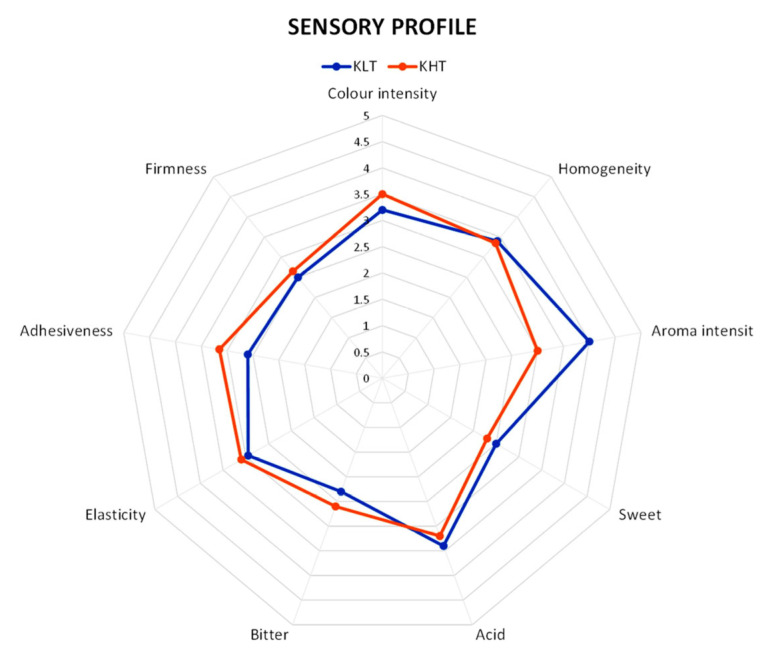
Sensory profile of kiwifruit slices dried at 40 °C (KLT) and 55 °C (KHT).

Sensory profiles for samples analyzed are shown in Figure 4. The main descriptors that showed higher scores were found to be color and aroma intensity. The overall results of the descriptive sensory analysis performed did not show significant differences among different treatments in most descriptors, except for the two main descriptors of color intensity, which was more intense in KLT samples and aroma intensity, that, on the other hand, was more intense in KHT samples; one textural descriptor (elasticity) was found to be ascribable to both statistical groups. No significant differences were found for taste descriptors.

Previously, Mahjoorian et al. [58] demonstrated that no significant differences were recorded on sensorial parameters such as color, odor, taste, and crunchiness (chewiness) (*p* < 0.01) in kiwifruits dried at temperatures from 50 to 70 °C. Furthermore, the sample that had the highest sensory score from a sensorial point of view was the one dried at 70 °C.

## 4. Conclusions

This study highlights, for the first time, the impact on chemical, sensorial, and health properties of dried Sungold cv. kiwifruit slices during conservation for 120 days in sealed containers in the dark at 25 °C. Slices were dried at two different temperatures, 40 and 55 °C, for 30 and 25 h, respectively, and monitored for their chemical-physical parameters, total bioactive compounds content, radical scavenging potential, sensory, and texture analysis.

The results obtained showed that regardless of the temperature applied, the obtained moisture content and water activity were suitable for preserving the dried samples from degradative reactions (chemical, enzymatic, or microbiological). Color was also affected by the drying process, although without significant differences. Textural parameters (hardness, elasticity, cohesiveness, rubberiness, and chewiness), apart from the resilience parameter result, showed significant differences between KLT and KHT samples, with values sometimes 2-times higher or more in the KLT samples compared to KHT samples. At the same time, a reduction in the content of bioactive compounds (vitamin C, phenolic and flavonoid compounds) and in the antioxidant activity was observed in proportion to the increase in drying temperature.

Regarding the sensory properties, it was possible to establish the sensory profiles of the dried samples, and the attributes that varied most among them were color and aroma intensity.

## Figures and Tables

**Table 1:** List of sensory descriptors for sample testing.

Category	Descriptor	Definition
Appearance	Color intensity	Overall product color intensity
	Homogeneity	Product shape perception
Olfactory	Aroma intensity	Overall product typical flavor intensity
Taste	Sweet	Overall product sweetness intensity
	Acid	Overall product acidity intensity
	Bitter	Overall product bitterness intensity
Texture	Elasticity	Springiness feeling intensity
	Adhesiveness	Mouth adhesivity feeling intensity
	Firmness	Structural firmness feeling intensity

**Table 2:** Chemical properties of kiwi slices after drying at different temperatures.

Days Storage	Moisture (wb)	a_w_	pH	TSS (°Brix)	TA (g Citric Acid/100 g dw)	BI
FKF						
T0	81.35 ± 2.32	0.99 ± 0.00	3.19 ± 0.04	14.50 ± 0.08	1.51 ± 0.00	
KLT						
T0	22.12 ± 0.23	0.45 ± 0.01 ^c^	3.56 ± 0.01 ^b^	2.43 ± 0.06 ^b^	0.74 ± 0.01 ^e^	50.57 ± 9.48
T30	22.09 ± 1.02	0.40 ± 0.00 ^d^	3.56 ± 0.01 ^b^	4.87 ± 0.12 ^a^	0.75 ± 0.01 ^a^	56.20 ± 14.38
T60	22.15 ± 1.25	0.47 ± 0.00 ^b^	3.51 ± 0.00 ^b^	4.37 ± 0.23 ^a^	0.68 ± 0.00 ^b^	55.07 ± 13.40
T90	22.21 ± 0.98	0.47 ± 0.00 ^b^	3.56 ± 0.02 ^b^	4.80 ± 0.00 ^a^	0.63 ± 0.03 ^c^	49.88 ± 10.20
T120	23.02 ± 2.06	0.52 ± 0.00 ^a^	3.70 ± 0.06 ^a^	4.27 ± 0.46 ^a^	0.58 ± 0.00 ^d^	49.92 ± 10.62
Sign.	ns	**	**	**	**	ns
KHT						
T0	19.21 ± 2.34	0.50 ± 0.00 ^ab^	3.52 ± 0.01 ^ab^	5.38 ± 0.03 ^a^	0.76 ± 0.07 ^b^	57.09 ± 8.11
T30	19.16 ± 1.98	0.44 ± 0.00 ^c^	3.54 ± 0.01 ^a^	3.5 ± 0.00 ^e^	0.69 ± 0.00 ^b^	55.00 ± 10.54
T60	19.13 ± 0.78	0.44 ± 0.00 c	3.37 ± 0.02 ^c^	5.17 ± 0.06 ^b^	0.93 ± 0.04 ^a^	57.22 ± 10.21
T90	19.27 ± 2.32	0.51 ± 0.00 ^a^	3.44 ± 0.00 ^cd^	4.03 ± 0.05 ^d^	0.73 ± 0.04 ^b^	46.29 ± 8.91
T120	19.87 ± 1.36	0.49 ± 0.00 ^b^	3.46 ± 0.05 ^c^	4.30 ± 0.00 ^c^	0.72 ± 0.00 ^b^	51.19 ± 10.91
Sign.	ns	**	**	**	**	ns

Fresh kiwifruits (FKF) and kiwifruit slices dried at 40 °C (KLT) and 55 °C (KHT). Data were expressed by means ± standard deviation (*n* = 3). Statistical analysis ANOVA were followed by Tukey’s test which were used to evaluate any differences at the same time of analysis. Result followed by letters are significant. ns: not significant; ** *p* ≤ 0.01.

**Table 3:** Color coordinates of kiwi slices after drying at different temperatures.

Days Storage	FKF			KLT			KHT		
	L	a*	b*	L	a*	b*	L	a*	b*
T0	58.57 ± 4.37	1.19 ± 0.78	15.34 ± 3.07	55.19 ± 2.85	7.45 ± 1.71 ^b^	18.74 ± 3.82	59.46 ± 3.74 ^ab^	7.09 ± 1.86	23.11 ± 3.60
T30				57.07 ± 4.29	8.61 ± 2.28 ^ab^	20.88 ± 5.11	57.19 ± 3.88 ^b^	6.54 ± 1.21	21.69 ± 4.92
T60				56.65 ± 3.78	9.04 ± 2.53 ^a^	20.29 ± 5.26	60.18 ± 3.85 ^a^	7.14 ± 1.24	23.54 ± 4.95
T90				55.60 ± 4.23	7.94 ± 1.86 ^ab^	18.28 ± 3.76	58.75 ± 3.58 ^ab^	7.39 ± 0.97	22.41 ± 4.25
T120				56.06 ± 4.12	7.99 ± 1.82 ^ab^	18.41 ± 3.85	57.87 ± 4.56 ^ab^	6.89 ± 1.50	20.39 ± 5.13
Sign.				ns	*	ns	*	ns	ns

Fresh kiwifruits (FKF), kiwifruit slices dried at 40 °C (KLT) and 55 °C (KHT). Data were expressed by means ± standard deviation (*n* = 3). Statistical analysis ANOVA were followed by Tukey’s test which were used to evaluate any differences at the same time of analysis. Result followed by letters are significant. ns: not significant. * *p* ≤ 0.05.

**Table 4:** Evolution of organic acids in analyzed dried kiwifruit slices during storage for 120 days.

Days Storage	Ascorbic Acid (mg/100 g dw)	Citric Acid (mg/100 g dw)	Malic Acid (mg/100 g dw)	Oxalic Acid (mg/100 g dw)	Tartaric Acid (mg/100 g dw)
FKF					
T0	957.22 ± 5.70	2215.47 ± 4.02	638.53 ± 5.06	62.96 ± 0.82	674.98 ± 2.38
KLT					
T0	1009.74 ± 5.11 ^a^	4664.67 ± 5.13 ^d^	390.45 ± 0.59 ^a^	27.49 ± 0.59 ^a^	508.54 ± 0.85 ^a^
T30	924.98 ± 3.93 ^b^	3621.49 ± 3.97 ^e^	385.64 ± 1.23 ^b^	27.14 ± 2.14 ^a^	441.33 ± 1.58 ^b^
T60	363.54 ± 2.11 ^c^	1217.32 ± 5.49 ^b^	230.60 ± 0.91 ^c^	1.23 ± 0.11 ^b^	302.42 ± 0.51 ^c^
T90	352.06 ± 2.72 ^cd^	845.98 ± 1.86 ^c^	40.22 ± 1.12 ^d^	nd	32.59 ± 2.71 ^d^
T120	339.94 ± 1.03 ^e^	886.74 ± 5.52 ^a^	28.87 ± 0.53 ^d^	nd	nd
Sign.	**	**	**	**	**
KHT					
T0	444.95 ± 3.52 ^a^	2008.12 ± 5.65 ^a^	341.21 ± 7.37 ^a^	99.67 ± 0.80 ^a^	526.99 ± 2.46 ^a^
T30	427.30 ± 7.51 ^a^	1967.13 ± 10.30 ^b^	327.39 ± 1.99 ^b^	28.37 ± 0.84 ^b^	472.39 ± 2.64 ^bc^
T60	428.88 ± 1.97 ^b^	1335.82 ± 4.23 ^c^	274.16 ± 3.00 ^c^	31.79 ± 3.75 ^b^	355.87 ± 1.01 ^abc^
T90	419.78 ± 2.96 ^a^	124.35 ± 0.38 ^d^	30.56 ± 2.31 ^d^	32.77 ± 1.98 ^b^	118.98 ± 1.59 ^c^
T120	384.29 ± 6.56 ^c^	90.88 ± 1.89 ^e^	20.58 ± 0.47 ^d^	1.99 ± 0.19 ^c^	49.64 ± 3.37 ^bc^
Sign.	**	**	**	**	**

Fresh kiwifruits (FKF), kiwifruit slices dried at 40 °C (KLT) and 55 °C (KHT). Data were expressed by means ± standard deviation (*n* = 3). Statistical analysis ANOVA were followed by Tukey’s test which were used to evaluate any differences at the same time of analysis. Result followed by letters are significant. ** *p* ≤ 0.01; nd: not detected.

**Table 5:** Total polyphenol content, total flavonoid content, and radical scavenging activity of fresh and dried kiwi slices under storage.

Days Storage	TPC (mg GAE/100 g dw)	TFC (mg CTE/100 g dw)	DPPH (mmol Trolox/100 g dw)	ABTS (mmol Trolox/100 g dw)
FKF				
T0	941.79 ± 4.49	260.19 ± 6.22	1195.87 ± 15.19	56.05 ± 3.06
KLT				
T0	979.42 ± 2.40 ^a^	281.84 ± 2.17 ^a^	1657.62 ± 0.92 ^a^	64.68 ± 0.34 ^a^
T30	650.54 ± 2.32 ^b^	273.84 ± 2.04 ^b^	1318.95 ± 6.62 ^b^	61.49 ± 2.95 ^ab^
T60	586.64 ± 2.05 ^c^	243.64 ± 3.70 ^c^	1241.47 ± 1.39 ^c^	58.00 ± 0.82 ^c^
T90	495.14 ± 5.43 ^d^	180.05 ± 0.99 ^d^	1024.68 ± 4.37 ^d^	55.93 ± 3.76 ^c^
T120	395.34 ± 0.85 ^e^	113.93 ± 1.12 ^d^	996.79 ± 2.63 ^e^	42.29 ± 1.61 ^d^
Sign.	**	**	**	**
KHT				
T0	526.04 ± 2.40 ^a^	169.07 ± 5.27 ^a^	926.15 ± 2.75 ^a^	67.59 ± 1.68 ^a^
T30	472.27 ± 4.80 ^b^	166.37 ± 3.50 ^a^	891.13 ± 5.13 ^b^	52.70 ± 2.37 ^b^
T60	456.82 ± 3.18 ^c^	165.33 ± 2.78 ^a^	889.00 ± 2.95 ^b^	45.96 ± 0.24 ^c^
T90	453.15 ± 2.64 ^c^	116.60 ± 2.90 ^b^	859.32 ± 1.49 ^c^	39.54 ± 1.39 ^d^
T120	408.25 ± 2.11 ^d^	102.78 ± 1.61 ^c^	854.35 ± 1.30 ^c^	36.71 ± 0.52 ^d^
Sign.	**	**	**	**

Fresh kiwifruits (FKF), kiwifruit slices dried at 40 °C (KLT) and 55 °C (KHT). GAE: Gallic Acid Equivalent; CTE: Catechin Equivalents. Data were expressed by means ± standard deviation (*n* = 3). Statistical analysis ANOVA were followed by Tukey’s test which were used to evaluate any differences at the same time of analysis. Result followed by letters are significant. ns: not significant; ** *p* ≤ 0.01.

**Table 6:** Degradation kinetic of TPC, TFC, and ascorbic acid in kiwifruit slices during storage for 120 days.

Bioactives	Drying	Reaction Order	*K*-Value (min^−1^)	R^2^	Half-Life (t_1/2_) (Days)
TPC	KLT	1	0.0018	0.9074	385.080
	KHT	1	0.007	0.9527	99.021
TFC	KLT	0	0.6078	0.8243	1.1404
	KHT	0	1.432	0.9173	0.4840

TPC: Total phenols content; TFC: Total flavonoids content; Kiwifruit slices dried at 40 °C (KLT) and 55 °C (KHT).

**Table 7:** Textural parameters for kiwi slices after drying at different temperatures (40 and 55 °C).

Days Storage	Hardness	Springiness	Cohesiveness	Gumminess	Chewiness	Resilience
KLT						
T0	11,782.33 ± 8252.49 ^b^	0.835 ± 0.07 ^a^	0.656 ± 0.09 ^a^	7327.57 ± 5156.92 ^b^	5967.77 ± 4106.79 ^b^	0.289 ± 0.06
T30	28,983.86 ± 7512.27 ^a^	0.712 ± 0.06 ^b^	0.579 ± 0.04 ^b^	16,881.93 ± 4660.77 ^a^	12,051.43 ± 3611.15 ^a^	0.332 ± 0.05
T60	27,160.28 ± 7642.55 ^a^	0.798 ± 0.07 ^ab^	0.599 ± 0.04 ^ab^	16,403.81 ± 5150.93 ^a^	13,191.35 ± 4674.64 ^a^	0.349 ± 0.06
T90	30,177.77 ± 7607.16 ^a^	0.727 ± 0.11 ^b^	0.552 ± 0.06 ^b^	16,858.77 ± 5026.39 ^a^	12,243.89 ± 3473.21 ^a^	0.323 ± 0.06
T120	29,158.98 ± 8077.83 ^a^	0.772 ± 0.08 ^ab^	0.573 ± 0.03 ^b^	16,755.93 ± 4782.70 ^a^	12,784.67 ± 3317.61 ^a^	0.339 ± 0.04
Sign.	**	**	**	**	**	ns
KHT						
T0	5038.91 ± 4020.93 ^b^	1.088 ± 0.39 ^a^	0.722 ± 0.07 ^a^	3403.76 ± 2676.40 ^b^	3313.20 ± 2266.48 ^b^	0.251 ± 0.09
T30	15,228.75 ± 7873.64 ^a^	0.93 ± 0.22 ^ab^	0.661 ± 0.06 ^ab^	9923.41 ± 5039.80 ^a^	8553.76 ± 4357.73 ^a^	0.335 ± 0.06
T60	14,507.64 ± 5317.43 ^a^	0.85 ± 0.05 ^ab^	0.625 ± 0.06 ^b^	9104.83 ± 3585.54 ^a^	7755.64 ± 3225.34 ^a^	0.271 ± 0.08
T90	14,543.67 ± 5551.77 ^a^	0.818 ± 0.07 ^b^	0.647 ± 0.05 ^b^	9260.87 ± 3153.48 ^a^	7468.94 ± 2225.94 ^a^	0.302 ± 0.06
T120	16,346.33 ± 4903.62 ^a^	0.855 ± 0.07 ^ab^	0.628 ± 0.05 ^b^	10,296.85 ± 3215.58 ^a^	8772.78 ± 2658.17 ^a^	0.321 ± 0.06
Sign.	**	*	**	**	**	ns

Kiwifruit slices dried at 40 °C (KLT) and 55 °C (KHT). Data were expressed by means ± standard deviation (*n* = 3). Statistical analysis ANOVA were followed by Tukey’s test, which were used to evaluate any differences at the same time of analysis. Result followed by letters are significant. ns: not significant; ** *p* ≤ 0.01.

## Data Availability

The original contributions presented in the study are included in the article/Supplementary Materials, further inquiries can be directed to the corresponding author.

## Supplementary Materials

The following supporting information can be downloaded at: https://www.mdpi.com/article/10.3390/foods13132100/s1, Table S1: Chroma (C) and Hue angle (h) of kiwi slices after drying at different temperatures; Table S2: Results of the descriptive sensory analysis; Table S3: Significant differences among the kiwi samples (KLT and KHT). Figure S1: Kinetics’ graphs. TPC (a) and TFC (b); Figure S2: Texture Profile Analysis (TPA) parameters; Figure S3: Texture Profile Analysis (TPA) calculations.
