# Supplementary material for: Evolution of Quality Parameters and Bioactivity of Actinidia chinensis cv. Sungold (Kiwifruit) Slices Subjected to Different Drying Conditions Storage for 4 Months
Source: Foods. 2024 Jul 1;13(13):2100. doi: 10.3390/foods13132100 (PMC11241542; doi:10.3390/foods13132100)
Supplement: Supplementary file 1 [file foods-13-02100-s001.zip › foods-3048926-supplementary.pdf]

SUPPLEMENTARY MATERIAL

Table S1. Chroma (C) and Hue angle (h) of kiwi slices after drying at different temperatures.

	**C**			**h (°)**		
Day storage	FKF	KLT	KHT	FKF	KLT	KHT
T0	21.99 ±2.78	24.18±1.21	20.17±1.00	1.54 ±0.65	1.41±0.00	1.48±0.00
T30		22.65±2.03	22.59±2.03		1.40±0.00	1.48±0.00
T60		24.60±2.10	22.21±2.00		1.37±0.00	1.48±0.00
T90		23.60±2.23	19.93±1.86		1.38±0.00	1.46±0.00
T120		21.52±2.56	20.07±1.97		1.38±0.00	1.46±0.00
Sign.		**	**		*	*

Fresh kiwifruits (FKF), kiwifruits slices dried at 40 °C (KLT) and 55°C (KHT). Data was expressed by means ± standard deviation (n= 3). Statistical analysis ANOVA were followed by Tukey's test which were used to evaluate any differences at the same time of analysis. Result followed by letters are significant at $p \leq 0.05$.

Table S2. Results of the descriptive sensory analysis.

**Sensory attributes**	**KLT**	**KHT**	**Sign.**
Colour intensity	3.1±1,25[a]	3.5±1.28[b]	**
Homogeneity	3.4±1.05[a]	3.35±1.02[a]	ns
Aroma intensity	4.0±0.77[b]	3±0.56[a]	**
Sweet	2.5±0.65[a]	2.3±0.61[a]	ns
Acid	3.4±0.87[a]	3.2±0.73[a]	ns
Bitter	2.30±0.44[a]	2.60±0.48[a]	ns
Elasticity	2.95±0.62[a]	3.10±0.52[ab]	*
Adhesiveness	2.60±0.45[a]	3.15±0.68[b]	**
Firmness	2.5±0.39[a]	2.65±0.43[a]	ns

Kiwifruits slices dried at 40 °C (KLT) and 55°C (KHT). Data was expressed by means ± standard deviation (n= 3). Statistical analysis ANOVA were followed by Tukey's test which were used to evaluate any differences at the same time of analysis. Result followed by letters are significant at $p \leq 0.05$. ns: not significant.

Table S3. Significance differences among the kiwi samples (KLT and KHT).

	**a_w**	**pH**	**TA**	**TSS**	**BI**
			Sign.		
T0	p < 0.004	p < 0.021	p < 0.070	p < 0.000	p < 0.001
T30	p < 0.288	p < 0.070	p < 0.002	p < 0.000	p < 0.216
T60	p < 0.021	p < 0.000	p < 0.000	p < 0.000	p < 0.058
T90	p < 0.008	p < 0.000	p < 0.001	p < 0.000	p < 0.012
T120	p < 0.008	p < 0.000	p < 0.000	p < 0.021	p < 0.195
	**L**	a*	b*	**TPC**	**TFC**
			Sign		
T0	p < 0.000	p < 0.681	p < 0.000	p < 0.000	p < 0.000
T30	p < 0.067	p < 0.059	p < 0.000	p < 0.000	p < 0.000
T60	p < 0.000	p < 0.004	p < 0.000	p < 0.000	p < 0.000
T90	p < 0.000	p < 0.763	p < 0.000	p < 0.000	p < 0.567

T120	p < 0.000	p < 0.088	p < 0.000	p < 0.000	p < 0.000
	**DPPH**	**ABTS**	**Ascorbic acid**	**Citric acid**	**Malic acid**
			Sign		
T0	p < 0.000	p < 0.000	p < 0.000	p < 0.000	p < 0.000
T30	p < 0.000	p < 0.000	p < 0.000	p < 0.000	p < 0.000
T60	p < 0.000	p < 0.000	p < 0.000	p < 0.000	p < 0.000
T90	p < 0.000	p < 0.000	p < 0.000	p < 0.000	p < 0.000
T120	p < 0.000	p < 0.000	p < 0.000	p < 0.000	p < 0.000
	**Tartaric Acid**	**Ossalic Acid**	**Hardness**	**Springiness**	**Cohesiveness**
			Sign.		
T0	p < 0.000	p < 0.000	p < 0.000	p < 0.000	p < 0.000
T30	p < 0.000	p < 0.000	p < 0.000	p < 0.000	p < 0.000
T60	p < 0.000	p < 0.000	p < 0.000	p < 0.000	p < 0.000
T90	p < 0.000	p < 0.000	p < 0.000	p < 0.000	p < 0.000
T120	p < 0.000	p < 0.000	p < 0.000	p < 0.000	p < 0.000
	**Gumminess**	**Chewiness**	**Resilience**		
		**Sign.**			
T0	p < 0.000	p < 0.000	p < 0.000		
T30	p < 0.000	p < 0.000	p < 0.000		
T60	p < 0.000	p < 0.000	p < 0.000		
T90	p < 0.000	p < 0.000	p < 0.000		
T120	p < 0.000	p < 0.000	p < 0.000		

Kiwifruits slices dried at 40 °C (KLT) and 55°C (KHT). TA: total acidity; TSS: Total Soluble Solid; BI:Browning index; TPC: Total phenols content; TFC: Total flavonoids content.

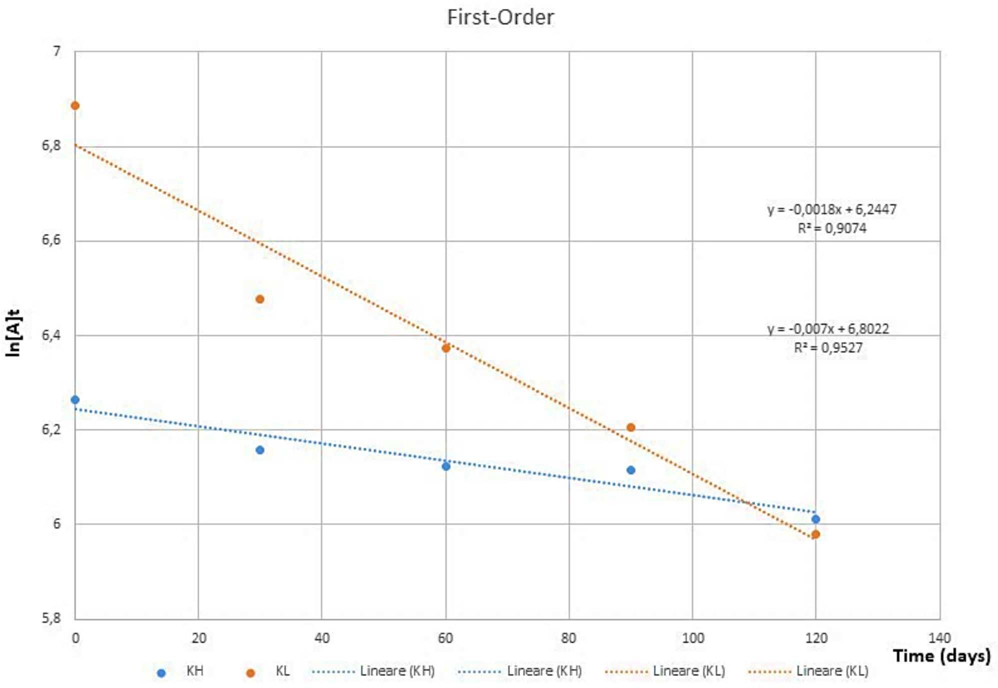

ln= natural logarithm

[A]= TPC concentration (GAE g 100 g^{-1})

(*a*)

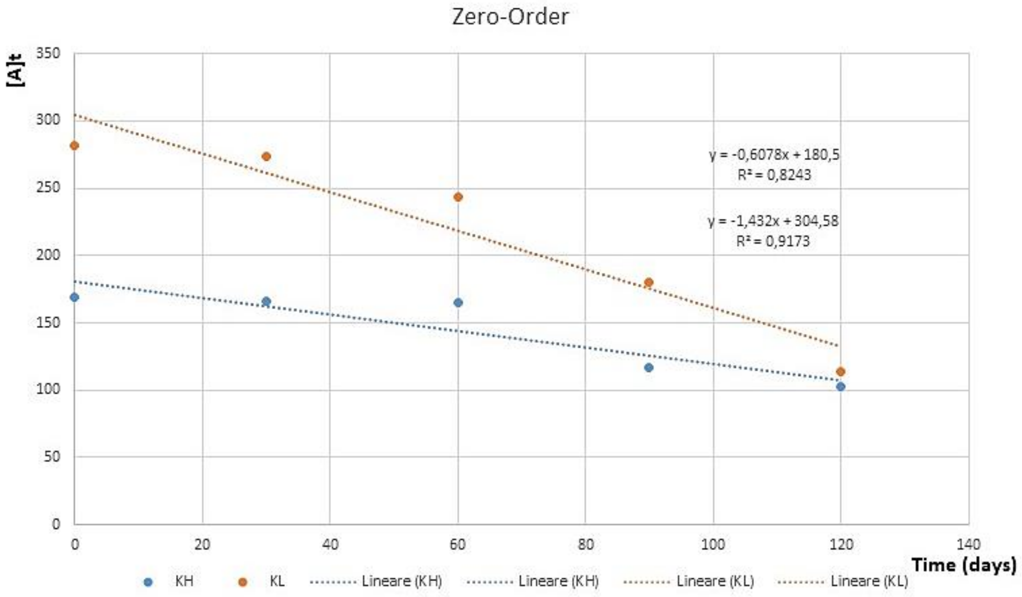

[A]= TFC concentration (CTE g 100 g^{-1})

(*b*)

Figure S1. Kinetics' graphs. TPC (*a*) and TFC (*b*).

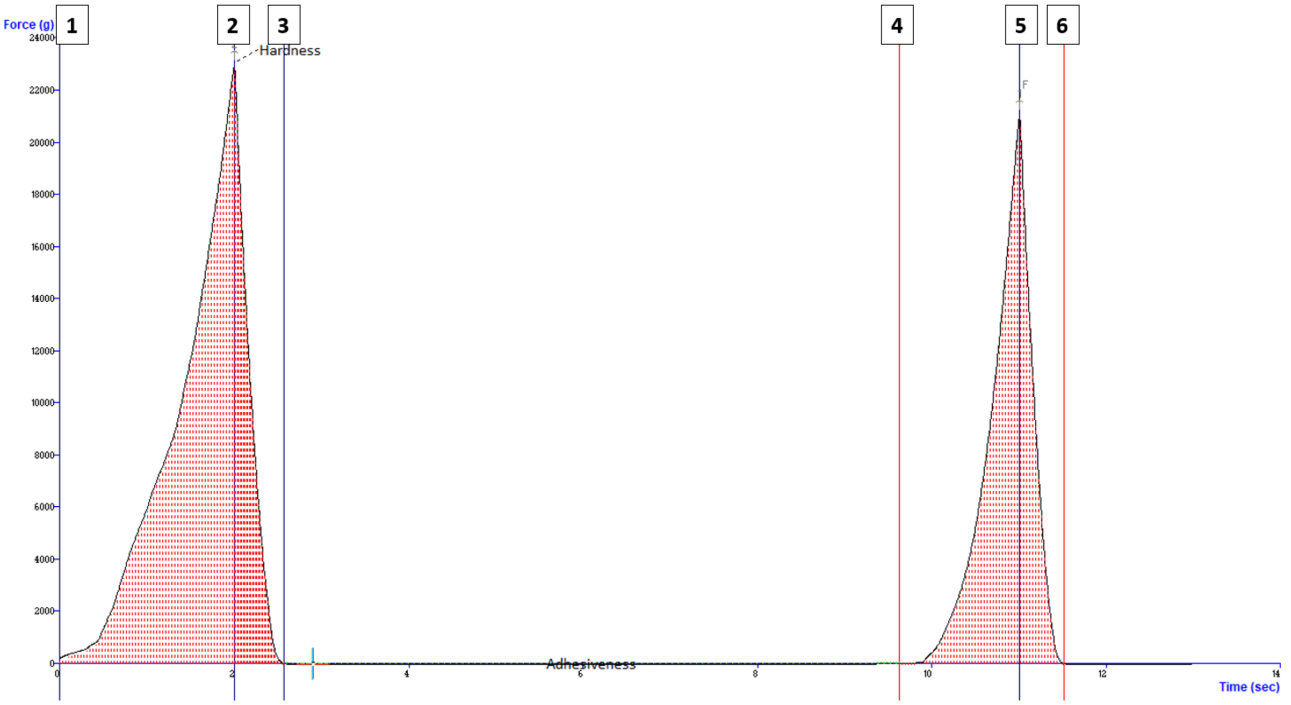

Explanation of TPA Parameters

Hardness: maximum peak force during the first compression cycle (Peak Force 2) (g)

Springiness: sample height recovered by sample between the end of the first bite and the start of the second bite (Ratio Time diff 4-5 / Time diff 1-2)

Cohesiveness: ratio of the positive force area during the second compression to that during the first compression (Ratio Area 4-6 / Area 1-3) adimensionale

Gumminess: product of hardness x cohesiveness (g)

Chewiness: product of gumminess x springiness (g)

Resilience: sample recovery from deformation both in terms of speed and forces derived (Ratio Area 2-3 / Area 1-2)

Figure S2. Texture Profile Analysis (TPA) parameters.

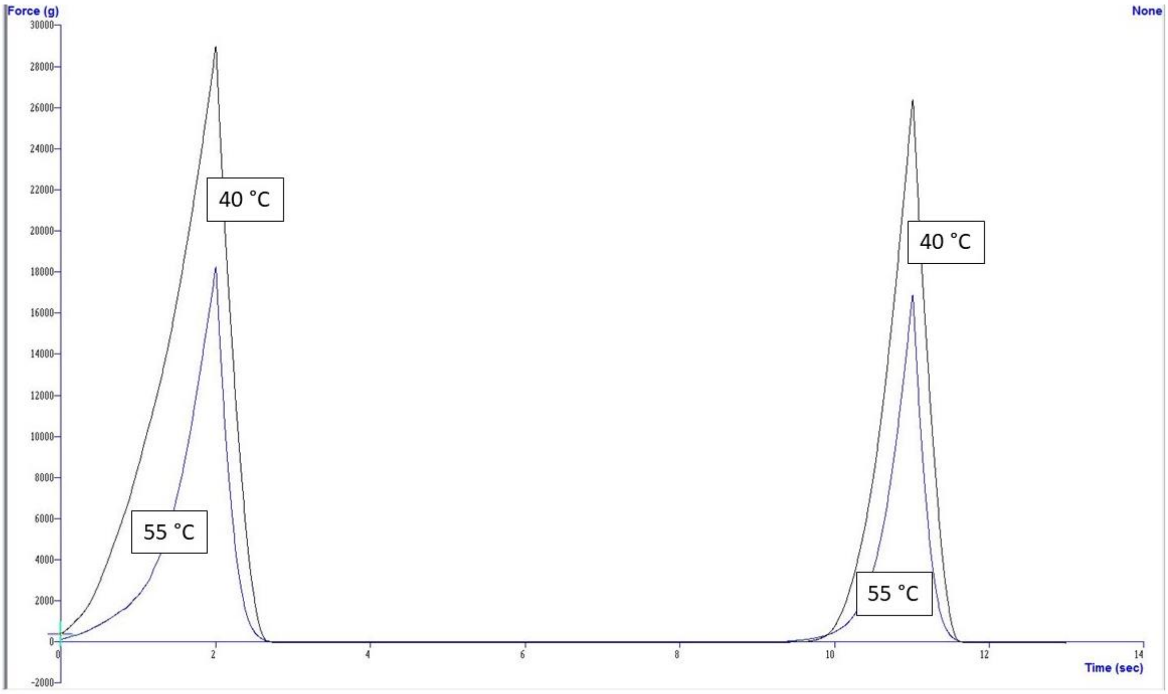

Figure S3. Texture Profile Analysis (TPA) calculations.
